# Dose-escalated salvage radiotherapy after radical prostatectomy in high risk prostate cancer patients without hormone therapy: outcome, prognostic factors and late toxicity

**DOI:** 10.1186/1748-717X-8-276

**Published:** 2013-11-27

**Authors:** Mohamed Shelan, Yasser Abo-Madyan, Grit Welzel, Christian Bolenz, Julia Kosakowski, Nadim Behnam, Frederik Wenz, Frank Lohr

**Affiliations:** 1 Department of Radiation Oncology, University Medical Center Mannheim, University of Heidelberg, Mannheim, Germany; 2 Department of Radiation Oncology and Nuclear Medicine (NEMROCK), Faculty of Medicine, Cairo University, Cairo, Egypt; 3 Department of Urology, University Medical Center Mannheim, University of Heidelberg, Mannheim, Germany

**Keywords:** Radical prostatectomy, Salvage radiotherapy, Dose escalation

## Abstract

**Purpose:**

Evaluation of dose escalated salvage radiotherapy (SRT) in patients after radical prostatectomy (RP) who had never received antihormonal therapy. To investigate prognostic factors of the outcome of SRT and to analyze which patient subsets benefit most from dose escalation.

**Materials and methods:**

Between 2002 and 2008, 76 patients were treated in three different dose-groups: an earlier cohort treated with 66 Gy irrespective of pre-RT-characteristics and two later cohorts treated with 70 Gy or 75 Gy depending on pre-RT-characteristics. Biochemical-relapse-free-survival (bRFS), clinical-relapse-free-survival (cRFS) and late toxicity were evaluated.

**Results:**

Four-year bRFS and cRFS were 62.5% and 85%. Gleason score <8, positive surgical resection margin (PSRM) and low PSA (≤0.5 ng/ml) before SRT resulted in higher bRFS. Analysis of the whole group showed no clear dose-outcome relationship. Patients with PSRM, however, had improved bRFS when escalating >66 Gy. While > 70 Gy did not improve the overall results, 4-year bRFS for patients with manifest local recurrence in the high-dose group was still comparable to those without manifest local recurrences. No grade 4 and minimal grade 3 gastrointestinal and urinary toxicity were observed.

**Conclusions:**

Dose-escalated SRT achieves high biochemical control. The data strongly support the application of at least 70 Gy rather than 66 Gy. They do not prove positive effects of doses >70 Gy but do not disprove them as these doses were only applied to an unfavorable patients selection.

## Background

Radical prostatectomy (RP) is an effective and widely employed curative treatment for localized prostate cancer. Despite that, biochemical relapse after RP will occur in 17–64% of the patients depending upon the selection criteria used, and up to a third of these patients will clinically progress to develop metastatic disease and ultimately die of prostate cancer [1].

Serum prostate-specific antigen (PSA) level after RP is a sensitive marker for tumor persistence and precedes clinical failure by years before the location of the recurrence becomes clinically evident [2]. Postoperative and/or salvage radiotherapy (SRT) offer a potentially curative treatment for selected patients with biochemical or local failure after RP and could reduce the risk of initial failure in high-risk patients [3,4]. The extent of postoperative and salvage radiotherapy and its most useful timing are frequently debated issues, with the line of separation between these two paradigms being increasingly blurred due to the advent of sensitive PSA-assays [5,6].

The optimal postoperative and salvage radiotherapy doses have not been defined. In the consensus statements on radiation therapy of prostate cancer, the American Society of Radiation Oncology (ASTRO) recommended a dose of “64 Gy or slightly higher”. In analogy to the treatment of primary tumors, delivering higher doses of radiotherapy to the prostatic bed without increasing morbidity became a possibility with the development of new radiotherapy techniques in the last few years. The impact of dose escalation in this situation is, however, not yet clear.

In this retrospective analysis, we evaluated the effect of dose-escalation on biochemical control in patients primarily treated with salvage intention radiotherapy) who had never received hormonal therapy prior to salvage RT. Further analysis aimed to evaluate the different prognostic factors related to the success of SRT and late treatment side effects of high dose salvage RT.

## Methods and materials

### Patient population

We evaluated the patients referred to Department of Radiation Oncology, University Medical Center Mannheim, to receive SRT after RP between 2002 and 2008. Analysis of the medical records was performed after approval by the ethics committee of the Medical Faculty Mannheim, University of Heidelberg. We reviewed the pre- and postoperative medical records, including surgical reports, pathology results, follow-up PSA values and the reported late toxicities.

### Patient characteristics

To study the efficacy of SRT alone without the influence of confounding therapies in a patient population without clinically overt metastases, we excluded the patients who had evidence of lymph node or distant metastasis (more than 80% of patients had a lymphadenectomy at primary surgery and are therefore pN0) and those who received any form of hormonal therapy either before or concomitantly with radiotherapy or after RT without documented PSA progression. Seventy six patients were included in this analysis.

### Treatment characteristics

Three different patient cohorts have to be looked at separately. Between 2002 and 2005, patients were treated with a 3D conformal technique with a dose of 66 Gy regardless of the risk factors (Group A). After this era the treatment concept was modified, identifying two patient strata: Patients who failed to achieve postoperative nadir (PSA nadir < 0.1 ng/ml) or had evidence of postoperative biochemical failure after RP (PSA rise above 0.2 ng/ ml) received a dose between 66 and 70 Gy (Group B). Patients who had positive resection margins and those who had radiological or histological evidence of local recurrence received a dose ≥ 70 Gy (typically ~75 Gy, ~15 Gy of which were applied in single doses of ~3 Gy as a hypofractionated boost to the volume with the highest presumed tumor cell density, Group C). A deviation from this approach occurred in two instances only: Two patients who had evidence of manifest local recurrence were treated nevertheless within group B at the responsible physician’s discretion.

Clinical target volume (CTV) included the prostate bed, the bladder neck, the urethral anastomosis and the seminal vesicle bed. The planning target volume (PTV) included the CTV with a margin of approximately 0.8 cm in all directions. Salvage RT was delivered using photon beams using 3D-conformal RT exclusively in 34% and a combination of 3D-conformal and Intensity modulated radiotherapy technique (IMRT) in 66%. The median radiation dose for all patients was 70 Gy (Range 66–75 Gy). Median dose was 66 Gy (Range 66–66) in group A, 69.1 Gy (Range 68–69.9 Gy) in Group B and 74.3 Gy (Range 70–75 Gy) in group C.

### Statistical analysis

A second biochemical PSA relapse after SRT was defined as a single PSA value >0.2 ng/ml higher than the post-radiotherapy nadir. Clinical progression was defined as the occurrence of a local relapse, lymph node metastasis or haematogenous metastasis.

Biochemical relapse free survival (bRFS) was calculated from the date of start of SRT until biochemical relapse or death. Clinical relapse free survival (cRFS) was calculated from the date of start of SRT to the date of clinical progression or death. The Kaplan-Meier method was used to estimate bRFS and cRFS. Univariate (log-rank) analysis was used to test the predictive value of patient-, tumor- and treatment-related factors on the bRFS. The p-value was considered significant for p ≤ 0.05.

### Toxicity

Any persisting chronic or late toxicities were considered after 6 months from the end of radiotherapy till the date of last follow up. All the data were tabulated and scored according to the LENT-SOMA toxicity scale.

## Results

Of 76 patients included in this analysis, 68 patients received SRT for rising PSA after RP (minimal time between RP and SRT: 4 months) and 8 patients were irradiated based on a positive surgical resection margin (PSRM) situation with either detectable or even rising PSA after surgery (minimal time between RP and SRT: 2 months). The patient- and tumor characteristics are summarized in Table 1.

**Table 1:** Patient and tumor characteristics

	**Group A**	**Group B**	**Group C**	**Total**
**Factor**	**66 Gy**	**> 66 Gy < 70 Gy**	**≥ 70 Gy**	
	**n =23 (%)**	**n = 15 (%)**	**n = 38 (%)**	**N = 76 (%)**
Age:				
< 60 years	10(43.5)	4(26.7)	10(26.3)	24(31.6)
≥ 60 years	13(56.5)	11(73.3)	28(73.7)	52(68.4)
Median				63
Preoperative PSA:				
≤ 10 ng/ml	10(43.5)	9(60)	22(57.9)	41(53.9)
>10 ng/ml ≤ 20 ng/ml	7(30.4)	4(26.7)	10(26.3)	21(27.7)
> 20 ng/ml	2(8.7)	0(0)	4(10.5)	6(7.9)
Missing	4(17.4)	2(13.3)	2(5.3)	8(10.5)
Median				7,9
Tumor size:				
pT2	8(34.8)	9(60)	19(50)	36(47.3)
pT3a	8(34.8)	3(20)	12(31.6)	23(30.3)
pT3b	6(26.1)	3(20)	7(18.4)	16(21.1)
Missing	1(4.3)	0(0)	0(0)	1(1.3)
Gleason score:				
≤ 6	4(17.4)	1(6.7)	2(5.3)	7(9.2)
7	13(56.6)	9(60)	23(60.5)	45(59.2)
**≥** 8	5(21.7)	5(33.3)	13(34.2)	23(30.3)
Missing	1(4.3)	0(0)	0(0)	1(1.3)
Surgical margin:				
R1	9(39.1)	5(33.3)	17(44.7)	31(40.7)
Rx (close)	4(17.4)	3(20)	9(23.7)	16(21.1)
R0	8(34.8)	7(46.7)	10(26.3)	25(32.9)
Missing	2(8.7)	0(0)	2(5.3)	4(5.3)
Risk groups:				
High	17(73.9)	13(86.7)	33(86.8)	63(82.9)
Intermediate	6(26.1)	2(13.3)	5(13.2)	13(17.1)
Pre-irradiation PSA:				
≤ 0.5 ng/ml	15(65.2)	9(60)	22(57.9)	46(60.5)
> 0.5 ng/ml	8(34.8)	6(40)	16(42.1)	30(39.5)
Median				0,5
Evidence of local recurrence				
Yes	2(8.7)	2(13.3)	17(44.7)	21(27.6)
No	21(91.3)	13(86.7)	21(55.3)	55(72.4)

**Figure 1:**
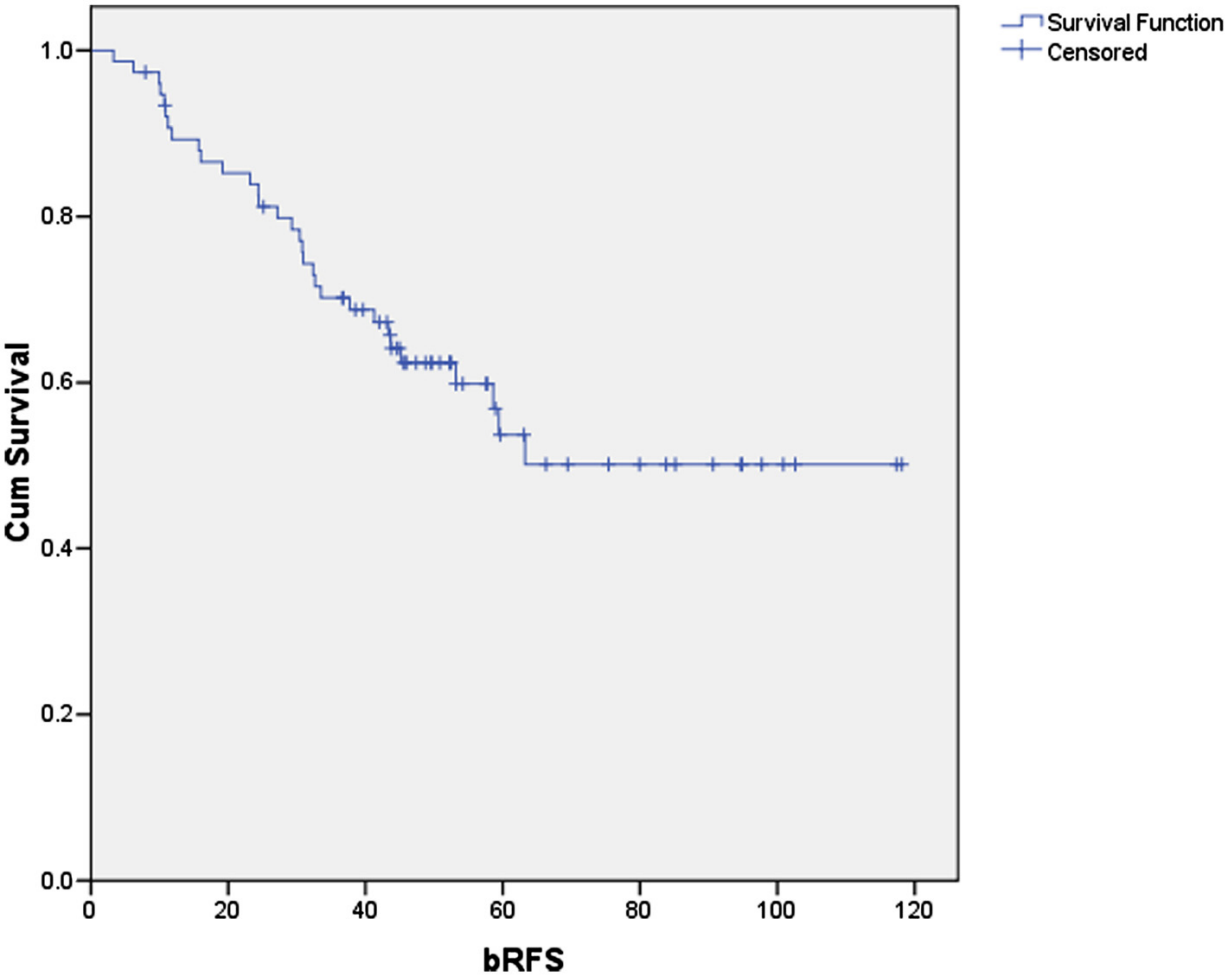
Kaplan-Meier curve for the biochemical relapse free survival (bRFS).

The eight-year overall actuarial survival was 85.6% and 11 patients were dead upon analysis. Only five patients were known to have evidence of distant metastasis at the time of death. Median follow-up after SRT was 52 months. Thirty one patients had a biochemical relapse and 13 patients developed distant metastases resulting in a 4 year bRFS of 62.5% (Figure 1) and a 4 year cRFS of 85%.

**Figure 2:**
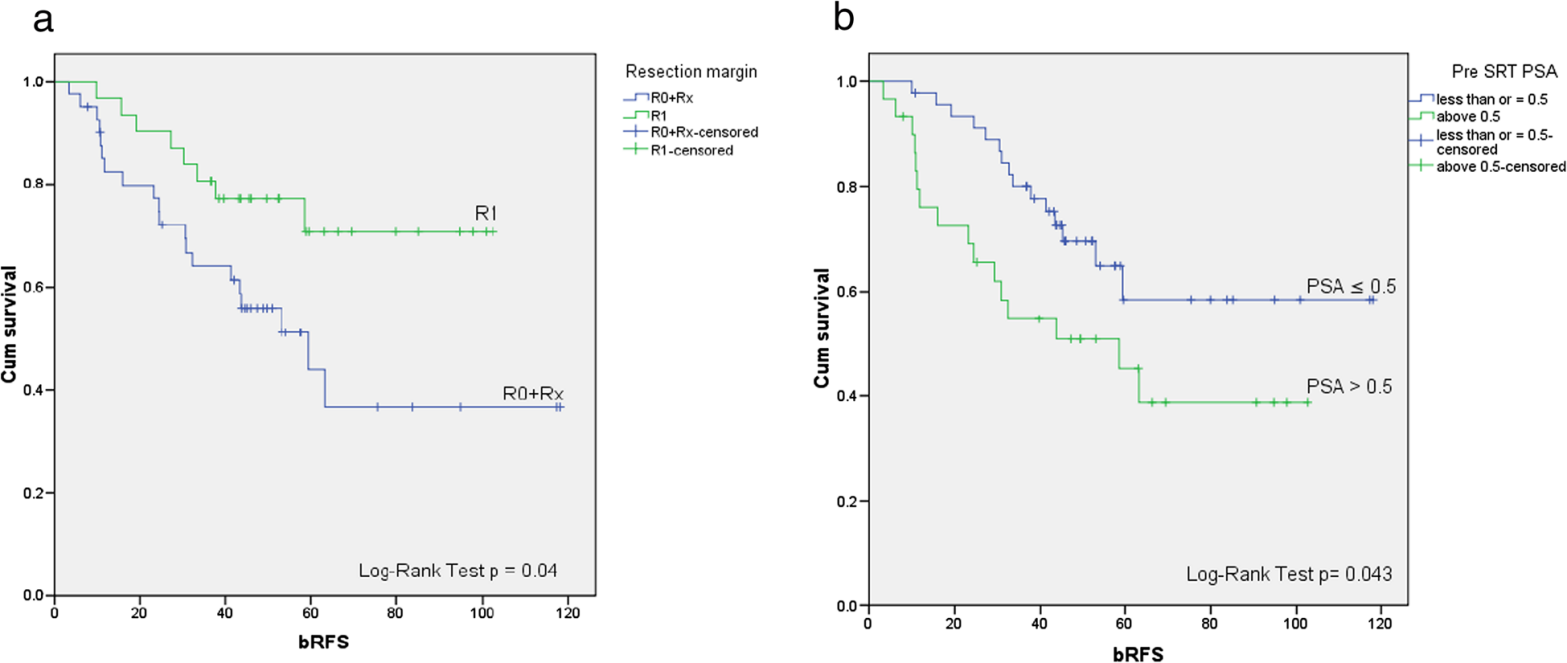
Kaplan-Meier curves for the effect of resection margin status on bRFS (a) and pre-irradiation PSA value on bRFS (b).

Upon univariate analysis a Gleason score of less than 8 (p = 0.039), a PSRM (p = 0.04) (Figure 2a) and a low PSA level (≤0.5 ng/ml) before SRT (p = 0.043) (Figure 2b) were associated with a significantly better bRFS (Table 2).

**Table 2:** Results of univariate analysis

**Factor**		**48 months bRFS%**	**p-value**
**Tumor size**	T2	71	0.2
	T3, T4	54	
**Gleason score**	≤ 7	70	**0.039**
	> 7	44.2	
**Initial PSA**	≤ 10	60	0.4
	>10-20	73.2	
	>20	25	
**Resection margin**	R0 + Rx	55.8	**0.04**
	R1	77	
**Local recurrence**	Yes	61	0.2
	No	63	
**Pre-radiation PSA**	≤ 0.5	70	**0.043**
	> 0.5	51	
**Dose**	= 66 Gy	63.6	0.1
	> 66 Gy < 70 Gy	85.7	
	≥70 Gy	52.5	

**Figure 3:**
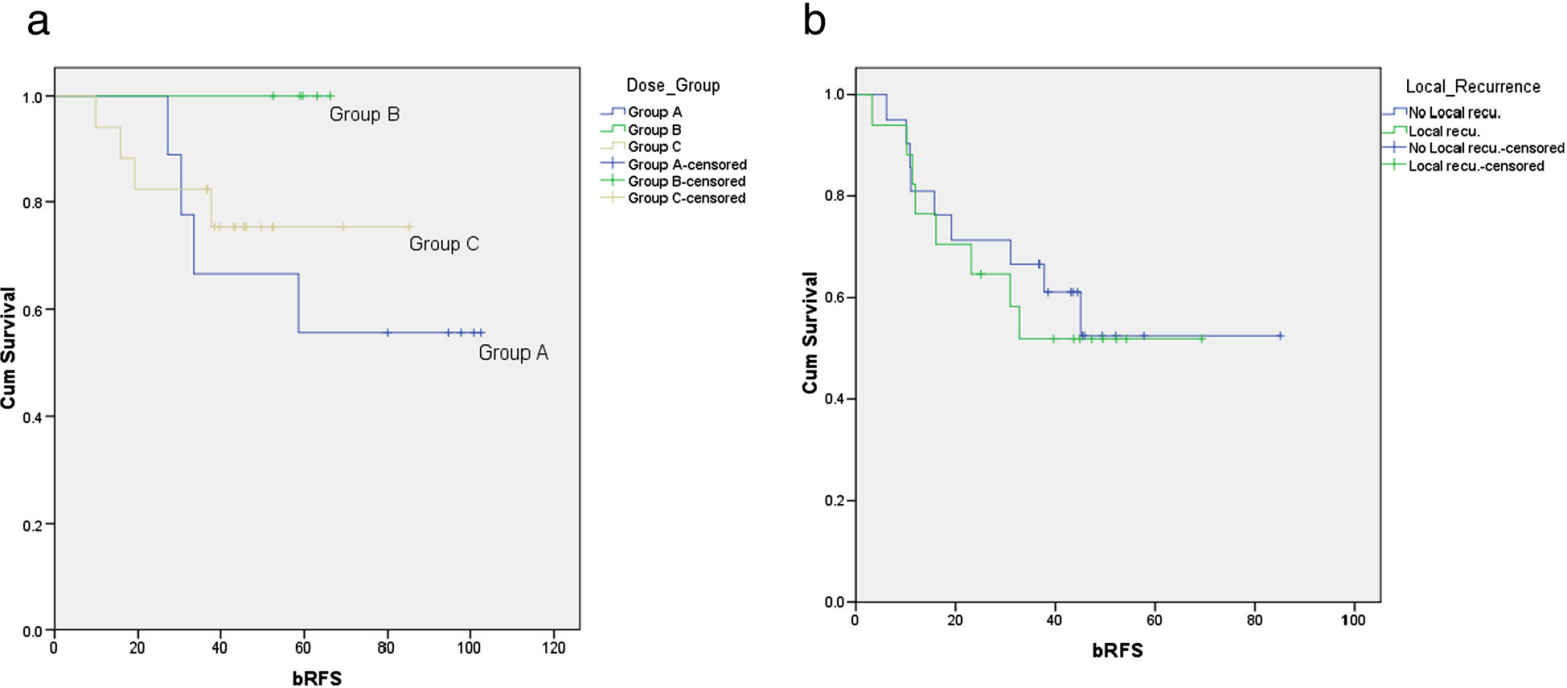
Kaplan-Meier curves for the bRFS for PSRM patients in the different dose groups (a) and patients with and without evidence of local recurrence in the group C (b).

When analyzing the whole patient pool, there was no clear relationship between biochemical outcome and dose. While this was not unexpected given the prospective stratification of dose based on perceived amount of tumor load for the patients treated later in the series, there were two cohorts that were sequentially comparable: patients with a positive resection margin in group B had higher bRFS than in group A (100% vs, 66.7%). bRFS for patients with positive resection margins in Group C (75.5%, the majority of which had manifest recurrence and therefore constituted a less favorable selection than those in group B) was lower than for those in group B but still higher than for those in group A (Figure 3a) therefore still suggesting a dose-effect relationship. In Group C, 4 year bRFS for the patients with manifest local recurrence was still comparable to bRFS for those who had no evidence of local recurrence (51.8% vs 52.4%).

### Toxicity

When classified according to LENT-SOMA, at the time of last follow up no grade 4 late urinary tract complications were reported. Grade 3 late urinary tract complications were observed in 3 patients who received a dose higher than 70 Gy and suffered from increased frequency. No Grade 3 or 4 late gastrointestinal complications were observed.

**Figure 4:**
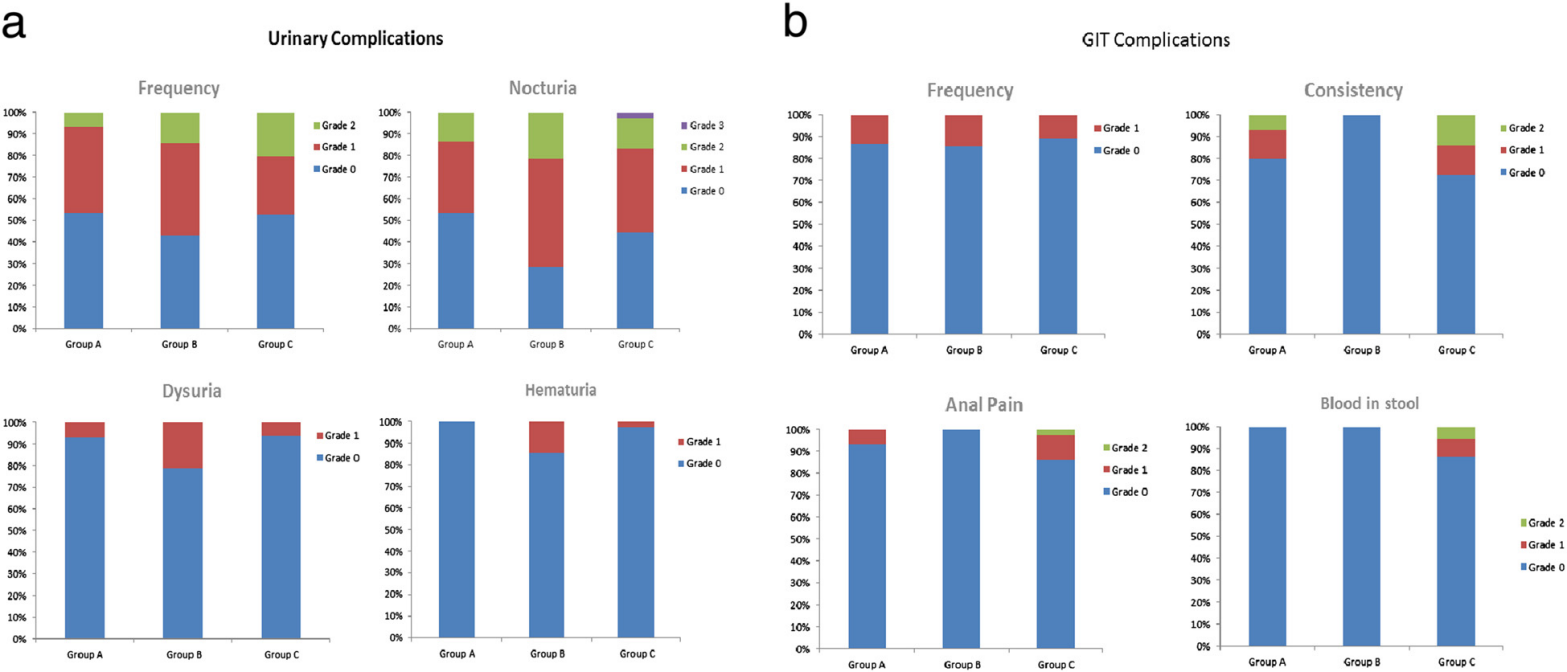
**The incidence of reported late urinary (4a) and gastrointestinal (4b) complications based on LENT SOMA toxicity scale in relation to the dose group (A: 66 Gy, B: >66 <70 Gy, C: ≥ 70 Gy).**≥** 70 Gy).**

Postoperative/pre-RT incontinence could not be assessed with certainty, therefore late incontinence was not assessed systematically. Figure 4 shows the incidence of reported late Urinary and Gastrointestinal complications in relation to the dose group.

## Discussion

The best treatment strategy for patients with a rise in postoperative PSA is still controversial. Salvage RT remains the only therapeutic option that offers a chance of cure in this situation [7]. Current diagnostic tools do not accurately identify the site of very early tumor recurrence. What is also unclear to date is the appropriate dose prescription, with current reports being in a corridor between 60 Gy and doses similar to what is applied in primary radical radiotherapy in excess of 70 Gy.

Several retrospective studies evaluated the efficacy of SRT in achieving biochemical control after post-RP relapse. Actuarial 5-year-bRFS rates range from 10% to 66% but methodologically it is difficult to compare between studies. Different prognostic factors related to a lower bRFS included: higher Gleason score, capsular or seminal vesicle extension, free-surgical margins, short PSA-doubling time and high pre-radiotherapy PSA level [8-10]. However, the previously mentioned factors were not consistently reported or correlated with treatment outcome among published studies. Pre-radiotherapy PSA level is the most consistent variable predicting bRFS. The largest analysis focusing on prognostic factors following SRT was a multi-institutional, retrospective cohort of 1540 patients who were treated with SRT between 1987 and 2005 with a median follow up of 53 months excluding patients who received additional hormonal therapy. The overall 6-year progression-free probability was 32%. When, however, patients were treated at serum PSA levels of less than 0.5 ng/ml, the progression-free survival probability was 48%, as compared with 18% if treated when the serum PSA level was greater than 1.5 ng/ml [8]. In another retrospective analysis of 635 patients, prostate cancer–specific and overall survival were significantly improved in patients who received SRT within 2 years of biochemical recurrence among men with a PSA doubling time of less than 6 months, independent of other prognostic features such as pathological stage or Gleason score [11].

Although PSRM at RP have been demonstrated to be an important predictor of disease recurrence [12], they have also been associated with improved bRFS after SRT, with a patient with positive margins who relapses being more likely to benefit from SRT targeting the pelvic/prostate-bed tissues than a patient with negative margins, whose PSA failure possibly represents distant disease [10,13].

In our series, a Gleason Score ≤7, a positive resection margin (R1) and pre-radiotherapy PSA level ≤0.5 ng/ml were the most important factors related significantly to a higher biochemical control for the whole cohort. In the individual dose strata the previous factors, except for the resection margin status, did not result in a significant difference in bRFS which was not expected due to the small numbers in every dose group.

A relevant issue is the question of the appropriate treatment dose. The advent of IMRT and Image-guided radiation therapy (IGRT) has rendered the application of doses in the range of what is being applied in definitive radiotherapy of prostate cancer possible.

In 1999, the American Society of Radiation Oncology (ASTRO) recommended a dose of “64 Gy or slightly higher” for SRT. The European Association of Urology (EAU) guidelines also recommend a dose between 64–66 Gy. Most of the published series using doses <66 Gy resulted in 5 years bRFS of less than 50% [9,14-16]. Further dose escalation in SRT above 66 Gy has been retrospectively analyzed with evidence of significantly improved bRFS [17]. King et al. published the results of a retrospective study comparing the outcome of 38 patients treated with 60 Gy and 84 patients treated with 70 Gy [15]. The results showed a significantly improved 5-year bRFS from 25% to 58% with the higher dose of 70 Gy. Another dose response analysis for SRT by Bernard et al. showed that doses greater than 66.6 Gy produced a clear reduction in biochemical failure [14].

Another recent analysis by Siegmann et al. showed that patients with decreasing PSA during SRT after RP had excellent outcome after receiving 70.2 Gy with low toxicity. In retrospect, the results were better than achieved in a cohort that, irrespective of response, was treated with 66.6 Gy. While a superiority of 70 Gy over 66 Gy can not be postulated based on these data because of the selection bias inherent to the selection process for dose escalation, these data nevertheless confirm the potential of SRT in patients with localized, radiosensitive disease [13,18].

While our series did not allow the analysis of a possible dose-effect relationship across the whole patient pool due to risk-based treatment stratification for the patients treated later in the series, the results for the sequentially comparable cohorts with similar risk treated at different dose levels suggests that a dose of 66 Gy is not sufficient but at least 70 Gy should be aimed for. This confirms an analysis published by Tomita et al. [6] who reported good efficacy of only 60 Gy in patients with a low PSA (<0.5 ng/ml) at SRT, but who, nevertheless, see a dose–response effect of doses ≥60 Gy vs. <60 Gy and thus, too, conclude that their data suggest that “salvage RT at doses higher than 60 Gy may improve biochemical outcome even in patients with a pre-RT PSA value ≤0.5 ng/ml”. An effect beyond 70 Gy could not be proven in our series with a significant number of patients with a PSA > 0.5 ng/ml because patients treated with these doses had an overall unfavorable risk profile. The results for these patients are inferior to those in the 70 Gy Group but still good given that most of these patients had manifest tumor relapse while those others did not. A dose-effect relationship for such patients beyond 70 Gy can therefore not be excluded.

The study by Ohri et al. published in 2012 showed that the biochemical control rates following SRT increase with SRT dose and decrease with high pre-SRT PSA values [19]. It was also reported that severe late GI and GU toxicity rates increase with SRT dose [19]. In our analysis based on the LENT SOMA scale the reported toxicity was minor in all dose groups, most likely reflecting the improved treatment techniques during the course of dose escalation. Randomized trials such as SAKK 09/10 are needed to finally decide if dose escalation is beneficial and in which patient strata.

Addition of hormonal treatment may eventually increase the risk of bias in the reported results of SRT, so, it is important to emphasize that the patients in our study had never been exposed to hormonal treatment before or after surgery or concomitantly with radiation. The benefit of adding hormone therapy to SRT has been examined in some retrospective studies [20]. The initial results of the phase III clinical trial (RTOG-9601) published in abstract form, comparing RT (64.8 Gy) with RT plus 2 years of high dose bicalutamide (150 mg per day) showed that addition of the hormone treatment during and after RT significantly improved the freedom from PSA progression and reduced the incidence of distant metastasis [21]. The final results of this trial are still pending.

## Conclusion

Our study suggests that a biochemical control rate of 62.5% in at 48 months can be achieved with SRT using a dose ≥ 66 Gy without addition of androgen suppressing therapy. Pre-radiotherapy PSA level remains an important prognostic factor for the rate of biochemical control. As in most series, a positive resection margin is associated with good biochemical control rates after SRT. While this analysis could not prove a positive effect of dose escalation beyond 70 Gy, it does not disprove it as these doses were only applied to an unfavorable patient selection. The data strongly support, however, the application of at least 70 Gy rather than 66 Gy. As a consequence of modern treatment techniques, such doses can now be applied with only moderate toxicity also in the salvage setting.

## Abbreviations

ASTRO: American society of radiation oncology; bRFS: Biochemical relapse free survival; cRFS: Clinical relapse free survival; CTV: Clinical target volume; EAU: European association of urology; IGRT: Image-guided radiation therapy; IMRT: Intensity modulated radiotherapy technique; PSA: Prostate-specific antigen; PSRM: Positive surgical resection margin; PTV: Planning target volume; RP: Radical prostatectomy (RP); SRT: Salvage radiotherapy.

## Competing interests

The authors declare that they have no competing interest.

## Authors’ contributions

MS and YAM performed data collection and contributed to writing the final manuscript. JK and NB performed data collection. MS and GW performed the statistical analysis. CB and FW contributed to data interpretation, read and discussed the manuscript. FL conceived the analysis and contributed to writing the manuscript. All authors read and approved the final manuscript.
